# Use of nitrogen-fixing bacteria to improve agricultural productivity

**DOI:** 10.1186/1753-6561-8-S4-O23

**Published:** 2014-10-01

**Authors:** Emanuel Maltempi Souza, Leda Satie Chubatsu, Luciano F Huergo, Rose Monteiro, Doumit Camilios-Neto, Roseli Wassem, Fábio de Oliveira Pedrosa

**Affiliations:** 1 Departamento de Bioquímica e Biologia Molecular, UFPR,Curitiba, Paraná, Brazil; 2 Universidade Estadual de Londrina, Londrina, Paraná, Brazil

## 

Nitrogen-fixing, plant-growth promoting bacteria are arguably the biotechnological tool of highest potential to improve agricultural productivity in short term. Nitrogen fixation and phytohormone production by these bacteria have been considered the most important factors for plant growth promotion. However, the underlying mechanisms responsible for productivity increases by associative bacteria are not clear. Moreover, the intensity of the plant growth promotion, including transfer of the fixed nitrogen from the bacteria to the plant, depends on an efficient interaction of the plant genotype and bacterial species. *Azospirillum* spp. are nitrogen-fixing, plant growth promoting bacteria that can associate with several cereals such as maize, rice and wheat, and also biofuel crops such as sugar cane and *Pennisetum. Azospirillum brasilense* is one of the most used plant growth promoting bacteria (PGPB), being used in Brazil, Argentina, Mexico, India and Europe. Analyses of field experiments have shown a success rate of inoculation with *Azospirillum* ranging from 60 to 70%, with statistically significant increases in yield varying from 5 to 30% [1]. The regulation of nitrogen metabolism in *A. brasilense* has been extensively studied. Here we will review nitrogen fixation regulation in this bacterium and advances in the understanding of aspects of interaction with cereal plants obtained by transcriptomic analyses.

## Regulation of nitrogen fixation in *A. brasilense*

The transcription initiation at *nif* promoters is dependent on the NifA protein. The function of NifA can be regulated at two levels, *nifA* gene transcription and NifA protein activity, and regulation at both levels occurs in response to oxygen and/or fixed nitrogen.

Evidence of a *nifA*-like gene *A. brasilense* was first obtained by Pedrosa and Yates (1984) by the isolation and characterization of a *nifA* mutant strain (FP10) [2]. The expression of the *nifA* gene in *A. brasilense* is only partially repressed by ammonium and oxygen, being maximal under nitrogen fixation conditions. In the absence of ammonium, oxygen levels have no effect on its expression, but it is partially repressed by ammonium under limiting oxygen conditions and almost fully repressed by high ammonium and high oxygen conditions. Analysis of the *nifA* promoter region showed two sequences resembling the Fnr consensus sequence located downstream from the probable transcription start, but their functionality has not been proved. Regulation of *nifA* expression seems similar to that *Sinorhizobium meliloti* in respect to oxygen repression and *Azorhizobium caulinodans* in respect to ammonium control of promoter activity. Indeed, there is evidence supporting the involvement of the two-component system FixJ-FixL and the FNR-like protein FixK in *A. brasilense*.

The activity of *A. brasilense* NifA protein is controlled by oxygen and ammonium concentrations. Oxygen-sensitivity of NifA is probably related to a linker region between the central and carboxy terminal domains containing a conserved Cys-X_4_-Cys, which together with 2 cysteine residues found in the central domain, form the cysteine motif Cys-X_11_-Cys-X_19_-Cys-X_4_-Cys potentially involved in binding an oxygen sensitive FeS cluster. Mutagenesis analysis showed that the cysteine motif is essential for the *B. japonicum* and *H. seropedicae* NifA activity *in vivo*.

A functional *glnB* gene is required for *A. brasilense* NifA activity. Since an N-terminal truncated NifA protein is active independently of the GlnB and is insensitive to the ammonium levels, regulation of NifA activity by GlnB probably involves its N-terminal domain. Furthermore, *A. brasilense* NifA is inactive in *E. coli*, but expression of *A. brasilense* GlnB is capable to activate NifA in this background. Moreover, purified GlnB and the N-terminal GAF domain are capable to interact *in vitro*.

The nitrogenase activity of *A. brasilense* is reversibly regulated by ADP ribosylation of the dinitrogenase reductase (NifH) in response to increase in ammonium concentration or energy depletion. This process is called nitrogenase switch-off and is catalysed by dinitrogenase reductase ADP-ribosyltransferase (DraT). Upon consumption of added ammonium or restoration of energy levels, the ADP-ribose group is removed from NifH by the dinitrogenase reductase activating glycohydrolase (DraG) leading to nitrogenase reactivation (nitrogenase switch-on). The two enzymes are oppositely regulated in response to the stimulus: under nitrogen fixing condition DraG is active and DraT is inactive, while addition of ammonium leads to activation of DraT and inactivation of DraG. A model for the ammonium-dependent control of DraT and DraG activities in *A. brasilense* has been proposed [3]. Under nitrogen fixing conditions the PII proteins GlnB and GlnZ are uridylylated and soluble in the cytosol. Under this condition DraG is active and DraT is inactive, therefore NifH is not modified and active. As ammonium levels increase, the PII proteins are deuridylylated by the uridylyl-removing enzyme GlnD. Deuridylylated GlnB stimulates the transferase activity of DraT triggering ADP-ribosylation of NifH with the consequent "switch-off" of nitrogenase activity. Deuridylylation of GlnZ causes the formation of DraG/GlnZ complex, which associates with the membrane protein AmtB, thereby removing DraG from the cytoplasm and inhibiting its ADP-ribosyl glicohydrolase activity [3].

This model can explain ammonium-induced switch-off of nitrogenase but the anaerobic induced NifH modification in *A. brasilense* is not dependent on GlnB, GlnZ or AmtB and must therefore occur by an independent mechanism.

## Transcriptomic analyses of wheat roots colonized by *A. brasilense*

*A. brasilense* is one of the most promising plant growth-promoting bacteria (PGPB). Wheat roots colonized by *A. brasilense* is a good model to investigate plant-PGPB interaction including improvement in plant growth. To comprehend the molecular basis of plant-*A. brasilense* interaction a RNA-Seq transcriptional profiling of wheat roots colonized by *A. brasilense* was carried out [4]. cDNA libraries from inoculated and non-inoculated wheat roots were sequenced and mapped to wheat EST database and *A. brasilense* genome. The unmapped reads were assembled *de novo*. A total of 23,215 expressed sequences from wheat were identified and 702 gene transcripts of *A. brasilense*. Bacterial colonization led to differential expression of 776 wheat ESTs belonging to various functional categories, including genes involved in transport activity, biological regulation, defense mechanism, production of phytohormones and phytochemicals. In addition, *A. brasilense* genes encoding proteins related to bacterial chemotaxis, biofilm formation and nitrogen fixation were highly expressed in colonized wheat roots. Interestingly, several ESTs encoding regulatory proteins of the wheat cell cycle were regulated by *A. brasilense*, which was consistent with a higher proportion of colonized root cells in the S-phase. The results reinforce that PGPB-inoculation might be an important alternative to improve nutrient acquisition, including nitrogen, and productivity in important crops such as wheat.

## Concluding remarks

The use of plant-growth promoting bacteria to improve productivity of important cereal crops such as maize and wheat is a reality. Research in this area has enhanced our understanding on the bacterial physiology and the mechanisms of bacteria-plant interaction. Among these bacteria, *A. brasilense* is one of most promising. Field experiments have shown consistent increase in productivity. Furthermore, *Azospirillum* is amenable to genetic studies and manipulation, and much is known about its nitrogen metabolism and regulation of nitrogen fixation. Finally, advances in omics sciences have allowed recent progress in the understanding of molecular aspects of plant-*Azospirillum* interaction. The knowledge accumulated on this system may allow future genetic manipulation to maximize the benefits of plant-growth promoting bacteria.
